# The Cell Envelope Structure of Cable Bacteria

**DOI:** 10.3389/fmicb.2018.03044

**Published:** 2018-12-20

**Authors:** Rob Cornelissen, Andreas Bøggild, Raghavendran Thiruvallur Eachambadi, Roman I. Koning, Anna Kremer, Silvia Hidalgo-Martinez, Eva-Maria Zetsche, Lars R. Damgaard, Robin Bonné, Jeroen Drijkoningen, Jeanine S. Geelhoed, Thomas Boesen, Henricus T. S. Boschker, Roland Valcke, Lars Peter Nielsen, Jan D'Haen, Jean V. Manca, Filip J. R. Meysman

**Affiliations:** ^1^X-LAB, Hasselt University, Hasselt, Belgium; ^2^Center for Electromicrobiology, Department of Bioscience Aarhus University, Aarhus, Denmark; ^3^Interdisciplinary Nanoscience Center and Department of Molecular Biology and Genetics, Structural Biology Aarhus University, Aarhus, Denmark; ^4^Department of Cell and Chemical Biology, Leiden University Medical Center, Leiden, Netherlands; ^5^Bio-imaging Core, Flemish Institute of Biotechnology (VIB), Gent, Belgium; ^6^Department of Biology, University of Antwerp, Antwerpen, Belgium; ^7^Department of Marine Sciences, University of Gothenburg, Gothenburg, Sweden; ^8^Department of Biotechnology, Delft University of Technology, Delft, Netherlands; ^9^Molecular and Physical Plant Physiology, Hasselt University, Hasselt, Belgium; ^10^Institute for Materials Research (IMO), Hasselt University, Hasselt, Belgium

**Keywords:** cable bacteria, long-distance electron transfer, cell envelope, periplasmic fibers, electron microscopy, atomic force microscopy

## Abstract

Cable bacteria are long, multicellular micro-organisms that are capable of transporting electrons from cell to cell along the longitudinal axis of their centimeter-long filaments. The conductive structures that mediate this long-distance electron transport are thought to be located in the cell envelope. Therefore, this study examines in detail the architecture of the cell envelope of cable bacterium filaments by combining different sample preparation methods (chemical fixation, resin-embedding, and cryo-fixation) with a portfolio of imaging techniques (scanning electron microscopy, transmission electron microscopy and tomography, focused ion beam scanning electron microscopy, and atomic force microscopy). We systematically imaged intact filaments with varying diameters. In addition, we investigated the periplasmic fiber sheath that remains after the cytoplasm and membranes were removed by chemical extraction. Based on these investigations, we present a quantitative structural model of a cable bacterium. Cable bacteria build their cell envelope by a parallel concatenation of ridge compartments that have a standard size. Larger diameter filaments simply incorporate more parallel ridge compartments. Each ridge compartment contains a ~50 nm diameter fiber in the periplasmic space. These fibers are continuous across cell-to-cell junctions, which display a conspicuous cartwheel structure that is likely made by invaginations of the outer cell membrane around the periplasmic fibers. The continuity of the periplasmic fibers across cells makes them a prime candidate for the sought-after electron conducting structure in cable bacteria.

## Introduction

Cable bacteria are long, multicellular, filamentous micro-organisms that are capable of generating and mediating electrical currents across centimeter-scale distances (Nielsen et al., 2010; Pfeffer et al., 2012; Meysman, 2017). They live in the surface layer of marine, freshwater, and aquifer sediments (Malkin et al., 2014; Risgaard-Petersen et al., 2015; Müller et al., 2016; Burdorf et al., 2017), and the presently known strains of cable bacteria belong to a narrow clade within the *Desulfobulbaceae* family of the Deltaproteobacteria (Trojan et al., 2016). Cable bacterium filaments consist of long, unbranched chains of cells that can extend up to 30–70 mm in length, and can include over 10^4^ cells in a single filament (Schauer et al., 2014). Cable bacteria also possess a gliding motility that helps them to orient themselves in the redox gradients that exist in aquatic sediments (Bjerg et al., 2016). In these sediments, they form dense networks, containing up to 2000 m of filament per cm2 of sediment surface (Malkin et al., 2014; Schauer et al., 2014; Vasquez-Cardenas et al., 2015), and their metabolic activity can exert a strong influence on the local ecosystem functioning and elemental cycling (Seitaj et al., 2015; Sulu-Gambari et al., 2016).

Still, the most conspicuous feature of cable bacteria is that they are capable of inducing long-distance electron transport. By transporting electrons from cell to cell along the longitudinal axis of their centimeter-long filamentous bodies, cable bacteria can utilize electron donors and electron acceptors in widely segregated locations, which provides them with a competitive advantage for survival in aquatic sediments (Nielsen and Risgaard-Petersen, 2015; Meysman, 2017). This process of long-distance electron transport overthrows some long-held ideas about energy metabolism and entails a whole new type of electrical cooperation between the cells of multicellular organisms (Meysman, 2017). Yet, at present, this process of long-distance electron transport remains highly enigmatic. Various lines of evidence indicate that the electrical current must be channeled through the cable bacterium filaments (Meysman, 2017). These include whole community perturbation experiments, in which the current stops upon a lateral cutting of the sediment (Pfeffer et al., 2012; Vasquez-Cardenas et al., 2015) as well as Raman microscopy of living individual filaments, which reveals a potential gradient along the filaments (Bjerg et al., 2018). Currently, neither the conductive structures nor the mechanism of electron conduction have been identified.

One hypothesis is that the conductive structures are located within the cell envelope of the cable bacterium filaments (Pfeffer et al., 2012). Scanning electron microscopy (SEM) has revealed that the outer surface of the filaments has an unusual topography, with parallel ridge compartments running along the whole length of the cable bacterium filaments (Pfeffer et al., 2012; Malkin et al., 2014). Subsequent electrostatic force microscopy measurements have shown a distinct elevation of the electrostatic force over the crest of the ridges, compared to the “valley” in between the ridge compartments, thus suggesting that the ridge compartments harbor a material with significant polarizability or charge storage capacity (Pfeffer et al., 2012). Based on these observations, the suggestion has been made that the ridge compartments of cable bacteria could harbor the conductive structures. This hypothesis warrants a closer investigation of the structural details of the cell envelope of cable bacteria. Here, we combined a range of microscopic techniques, including classical scanning electron microscopy (SEM), transmission electron microscopy (TEM), cryo-based electron microscopy (cryoEM) and tomography (cryoET), focused ion beam-scanning electron microscopy (FIB-SEM), and atomic force microscopy (AFM) on both intact cable bacteria and extracted fiber sheaths. The goal was to develop an improved structural model for the cell envelope of cable bacteria.

## Materials and Methods

### Cultivation and Specimens Preparation

#### Cable Bacteria Enrichment

A sediment enrichment procedure was implemented to produce cable bacterium filaments for microscopic investigation. Sediment was collected from different field sites where cable bacteria were previously demonstrated to be abundant *in situ*. Sampling locations included five marine sites (Lake Grevelingen, the Netherlands; Aarhus bay, Denmark; Rattekaai salt marsh, the Netherlands; Coastal zone Station 130, Belgium; Cocksdorp intertidal flat, the Netherlands) and one freshwater site (Aarhus University Lake, Denmark). This broad field survey allowed to assess the morphological diversity between filaments from different geographical locations, as well to identify the morphological features that are commonly shared by different cable bacterium strains. The sediment enrichment procedure has been described in detail elsewhere (Burdorf et al., 2017). Briefly, sediments were sieved, homogenized, repacked in PVC core liner tubes (diameter 40 mm, height 100 mm). Sediment cores were subsequently incubated under aerated conditions, and when the sediment showed the distinct geochemical fingerprint of electrogenic sulfur oxidation, as determined by high resolution microsensor profiling (O_2_, H_2_S, and pH), it was used for the retrieval of cable bacterium filaments. Unless stated otherwise, filaments were gently pulled from the top sediment layer with a thin glass needle (pulled from Pasteur pipets) under a binocular microscope at 8–50 × magnification, and then quickly transferred to a solution droplet on a carrier substrate for further sample preparation.

#### Washing Procedure to Obtain Filaments With Intact Cell Envelope Structure

The goal of the “washing procedure” was to obtain cable bacterium filaments that retain an intact cell envelope structure, but are free from sediment debris and do not show attached salt crystals resulting from salt precipitation upon drying. To this end, cable bacterium filaments were transferred from the sediment enrichments into a drop of purified water (ISO 3696 Grade 1, MilliQ) on a glass cover slip. Subsequently, small bundles of filaments were transferred between distinct droplets of MilliQ on the same cover slip. This way, filaments were exposed to 3–10 consecutive washes to remove sediment debris and salt, and the resulting specimens are referred to as “washed filaments.” MilliQ is a hypotonic solution, potentially causing osmotic lysis, and the impact of the MilliQ washing procedure on the structure of the filaments is discussed in detail below.

#### Extraction of the Periplasmic Fiber Sheath

The goal of the extraction procedure was to remove the cytoplasm and membranes, while retaining a periplasmic sheath structure that still included the tentative conductive fibers. To this end, filaments were subjected to a sequence of washes and chemical extractions immediately after retrieval from the sediment enrichment. In a first step, filaments were exposed to 3–10 consecutive washes in MilliQ on a glass cover slip, as already described above. Washed intact cable bacterium filaments were then incubated for 10 min at room temperature (RT) in a droplet of 1% (w/w) aqueous solution of sodium dodecyl sulfate (SDS), again followed by six MilliQ washes. Subsequently, specimens were transferred to a droplet of 1 mM aqueous solution of sodium ethylene diamine tetra acetate (EDTA), pH 8, incubated for 10 min at RT, and finally washed six times in MilliQ. The resulting specimens are referred to as “extracted filaments,” and the effects of the extraction procedure on the filaments is discussed in more detail below.

## Microscopy

### Scanning Electron Microscopy (SEM)

Specimens for low vacuum SEM were obtained on ACLAR® slides (Agar Scientific, United Kingdom) that were inserted into sediment enrichments. After a few days of incubation, the ACLAR® slides were carefully pulled from the sediment and gently washed with artificial seawater at *in situ* salinity (to prevent osmotic effects and lysis). After washing, a number of cable bacterium filaments remained firmly attached to the ACLAR® slides, while sediment and debris were removed. These slides were then transferred through a gradual ethanol gradient (25, 50, 70, 90%, 2 × 100%, for 10 min each), before being critical point dried with carbon dioxide (BALTEC CPD 300, Leica Microsystems, Wetzlar, Germany). Subsequently, specimens were mounted on aluminum stubs (diameter 12 mm), and coated with ~5 nm gold (JEOL JFC-1200 Fine Coater, JEOL, Tokyo, Japan). Images were made with a JEOL JSM-5600 LV (JEOL, Tokyo, Japan) under high vacuum operated at 10–30 kV.

### Transmission Electron Microscopy (TEM)

After initial sample preparation (washing or extraction procedure as described above), specimens were pipetted in a small volume of MilliQ water onto Formvar®-coated or carbon-coated copper grids and allowed to air-dry on the grids. The carbon-coated grids were subjected to glow discharge (15–25 mA, 45 s) before use. Samples were left either unstained or were stained by incubation in a 1% uranyl acetate solution for 45 s before drying. High vacuum TEM imaging was performed on a Tecnai Spirit Electron Microscope at 120 kV using a 4 × 4 k Eagle camera (Thermo Fischer Scientific, Waltham MA, USA) or at 80 kV using a 2 × 2 k Veleta side-mounted camera (Olympus SIS, Munster, Germany).

### Cryo-Imaging

Both cryo-electron microscopy (cryoEM) and its extension cryo-electron tomography (cryoET) were implemented. Individual filaments for cryoEM were pulled from the top sediment layer with a thin glass needle under a light microscope (8–50×; Zeiss Stemi SV6, Zeiss, Oberkochen, Germany), washed in artificial seawater at *in situ* salinity and transferred onto copper EM grids covered with a perforated carbon film (QUANTIFOIL®, Quantifoil Micro Tools, Germany) that were glow discharged in air (15–25 mA, 60 s; Emitech K950X, Quorum Emitech, London, United Kingdom). Specimens were vitrified (Leica EM GP, Leica Microsystems, Wetzlar, Germany), after 1–2 s blotting in a humidity chamber operated at 22°C and 100% humidity, by plunging them into a liquid ethane/propane mixture. For cryoET, 15 nm colloidal gold particles coupled to protein A (CMC, the Netherlands) were added to the samples as fiducial markers. CryoET imaging was performed on a Titan Krios™ (Thermo Fischer Scientific, formerly FEI Company, Waltham MA, USA) operated at 300 kV. Images were recorded using Explore 3D software on a 2 × 2 k CCD camera mounted behind a GIF energy filter (Gatan, Pleasanton CA, USA) operated at a slit width of 30 eV. Tomograms were recorded with 3° tilt steps between −51° to +51° at a defocus of −100 μm, at 4,800 × magnification (3.17 nm pixels size) and 2° tilt steps between −50° to +50°, −30 μm defocus and 8,700 × magnification (1.74 nm pixel size). The choice of these specific tomography settings—limited tilt, low magnification (4,800/7,800x) and high defocus (−100/−30 micron under focus)—was dictated by the large sample thickness of the cable bacterium filaments. Tomographic tilt series were processed using the software IMOD version 4.7 (Kremer et al., 1996) and 3D surface rendering was performed by manual segmentation using AMIRA software version 6.0 (Thermo Fischer Scientific, formerly FEI Company, Waltham MA, USA).

### Focused Ion Beam Scanning Electron Microscopy (FIB-SEM)

After initial sample preparation (washing or extraction procedure as described above), specimens for FIB-SEM were embedded in a thin layer of 2% low melting point agarose (Thermo Fischer Scientific, USA). Afterwards, the samples were incubated in freshly prepared fixative (2% paraformaldehyde, Applichem, Germany), 2.5% gluteraldehyde (EMS, USA) in 0.15 M sodium cacodylate (Sigma-Aldrich, USA) buffer with a pH 7.4 at RT for 30 min. Fixative was removed by washing 5 × 3 min in 0.15 M cacodylate buffer and samples were incubated in 1% osmium tetroxide (OsO_4_) (EMS, USA), 1.5% potassium ferrocyanide (Sigma-Aldrich, USA) in 0.15 M cacodylate buffer for 40 min at RT. This was immediately followed by a second incubation in OsO_4_ (1% OsO_4_ in double-distilled H_2_O) for 40 min at RT. After washing in double-distilled H_2_O for 5 × 3 min, samples were incubated overnight at 4°C in 1% uranyl acetate (EMS, USA). The next day, uranyl acetate was removed by washing in double-distilled H_2_O for 5 × 3 min. After the final washing steps the samples were dehydrated using solutions of increasing ethanol concentration (30, 50, 70, 90%, 2 × 100%), for 3 min each at 0°C. Subsequent infiltration with Durcupan™ resin (EMS, USA) was done by first incubating in 50% resin in ethanol for 2 h, followed by at least 3 changes of fresh 100% resin (including 1 overnight incubation). Finally, samples were embedded in fresh resin and cured in the oven at 65°C for 72 h. The resin block containing cable bacterium filaments was mounted on aluminum SEM stubs and samples were coated with ~6 nm of platinum (Quorum Q150T ES, Quorum Technologies, Laughton, United Kingdom). FIB-SEM imaging was performed using a Zeiss Auriga Crossbeam system (Zeiss, Oberkochen, Germany). The focused ion beam was set to remove 5 nm sections by propelling gallium ions at the surface. In between milling of sections, samples were imaged at 1.5 kV using an energy-selective back-scattered electron detector. This produces a stack of images which can then be further compiled into 3D data and processed using interactive stack rotation in the software Fiji/ImageJ (Schindelin et al., 2012; Schneider et al., 2012).

### Atomic Force Microscopy (AFM)

After initial sample preparation (washing or extraction procedure as described above), specimens were transferred onto mica substrates in a drop of MilliQ and left to dry in air. The mica substrates were subsequently glued to stainless steel discs of 12 or 15 mm diameter with silver paste. Images were collected using a Multimode 8 microscope (Bruker, Santa Clara CA, USA) in the peak-force quantum nano mechanical mode (PF-QNM) at ambient atmospheric pressure. The probes (TAP-150A, Bruker, Santa Clara CA, USA) contain a rectangular cantilever with a pyramidal tip with a nominal tip radius of 8 nm. The cantilever is made of antimony doped silicon, with a reflective aluminum coating to improve the laser signal by a factor of up to 2.5. AFM data were processed with Gwyddion software (Nečas and Klapetek, 2012), and topography images were flattened by fitting a plane over the mica substrate through three points. The limits of the color scale were adjusted for topography and PF-QNM images (these limits correspond to the minimum and maximum values on the color scale range). When required, a large image was cropped or an image was converted to a 3D view, in which dimension arrows were added using the program Autodesk® Inventor® 2018.

### Image Analysis

Image analysis was done in the Fiji/ImageJ software (Schindelin et al., 2012; Schneider et al., 2012). SEM images were analyzed to arrive at an estimate for the mean ridge width δ_*R*_, which is defined as the perimeter length attributed to a single ridge compartment of the cell envelope. The quantity *N*_*obs*_ denotes the number of ridges as counted from the SEM image. As only one side of a filament is visible in the SEM images, the total number of ridges *N*_*R*_ was estimated as *N*_*R*_ = 2 · *N*_*obs*_. As a result of sample preparation and preservation bacterial filaments can be flattened, and hence they no longer have a cylindrical geometry. The degree of flattening ε theoretically ranges between ε = 0 (perfectly flat) and ε = 1 (perfectly round). The filaments in our SEM images were assumed to have an ellipse cross-section, where *W* is the length of major axis and ε·*W* is the minor axis length. Hereby was W measured as the transverse cross-sectional width of the filament in the microscopy image. The flattening ε was estimated for each filament from the image context. The perimeter of the ellipse *P*_*SEM*_ was subsequently calculated by the Ramanujan approximation, where the auxiliary parameter *h* = (1 − ϵ)^2^/(1 + ϵ)^2^ was calculated to obtain the perimeter as

$$\begin{array}{l} P_{SEM}=\frac{\pi }{2}W\left(1+\varepsilon \right)\left(1+\frac{3h}{10+\sqrt{4-3h}}\right) \end{array}$$

For a perfectly round filament (ε = 1), this formula gives *P* = π*W*, while for a perfectly flat filament (ε = 0), *P* ≈ 2*W* is obtained. The average ridge width δ_*R*_ was finally determined as δ_*R*_ = *P/N*_*R*_ and equivalent spherical diameter as *d*_*ESD*_ = *P*/π.

For FIB-SEM images, the stacked images were rotated in ImageJ to arrive at transverse cross-sections through the filaments. From these, the ridge number *N*_*R*_ was obtained by counting the individual ridges and the perimeter *P* was derived by tracking the circumference of the cells. This was done at multiple cross-sections throughout a single filament. If a filament was only partially imaged, the arc length *L* was measured over an angle θ, and the perimeter was calculated as *P* = *L* · 2π/θ. The ridge width δ_*R*_ = *P /N*_*R*_ and equivalent spherical diameter as *d*_*ESD*_ = *P*/π were subsequently calculated. The ridge compartment area *A*_*R*_ was obtained by tracking the circumference of single ridge compartments at a cross-section of a cell. Similarly, the bulb diameter δ_*B*_, the bulb core diameter δ_*C*_ and the stalk width δ_*S*_ were obtained by measuring the width of these components at a junction cross-section (the meaning of these components is detailed below and indicated in **Figures 3G,H**). The bulb diameter was then used to calculate the bulb area $A_{B}=\pi {\left(\delta_{B}/2\right)}^{2}$. A reconstructed 3D cable bacterium model was build using Autodesk® Inventor® 2018.

In the AFM images, we observed that the air-dried filaments more strongly adhered to the mica substrate. Consequently we assumed that the filaments attained a half-ellipse shape with height *H* and a width *W* at the middle of the cell, and a flattening defined as ε = *2H/W*. Both *H* and *W* were calculated from the AFM images using Python software version 3.6.5 (Rossum 1995), and the perimeter *P* was calculated using the Ramanujan approximation as

$$\begin{array}{l} P=\frac{\pi }{2}\;\frac{W}{2}\left(1+\varepsilon \right)\left(1+\frac{3h}{10+\sqrt{4-3h}}\right)+W \end{array}$$

The equivalent spherical diameter was calculated as *d*_*ESD*_ = *P*/π. The ridge compartment width δ_*R*_ was measured as the distance between two ‘valleys' surrounding an individual ridge.

### Statistical Analysis

All analyses were performed in R version 3.5.0 (R Core Team, 2018) or Python version 3.6.5 (van Rossum, 1995). Values mentioned in the tables and text are averages ± one sample standard deviation (unless otherwise mentioned).

## Results

### SEM and TEM Imaging of Intact Filaments

**Figure 1:**
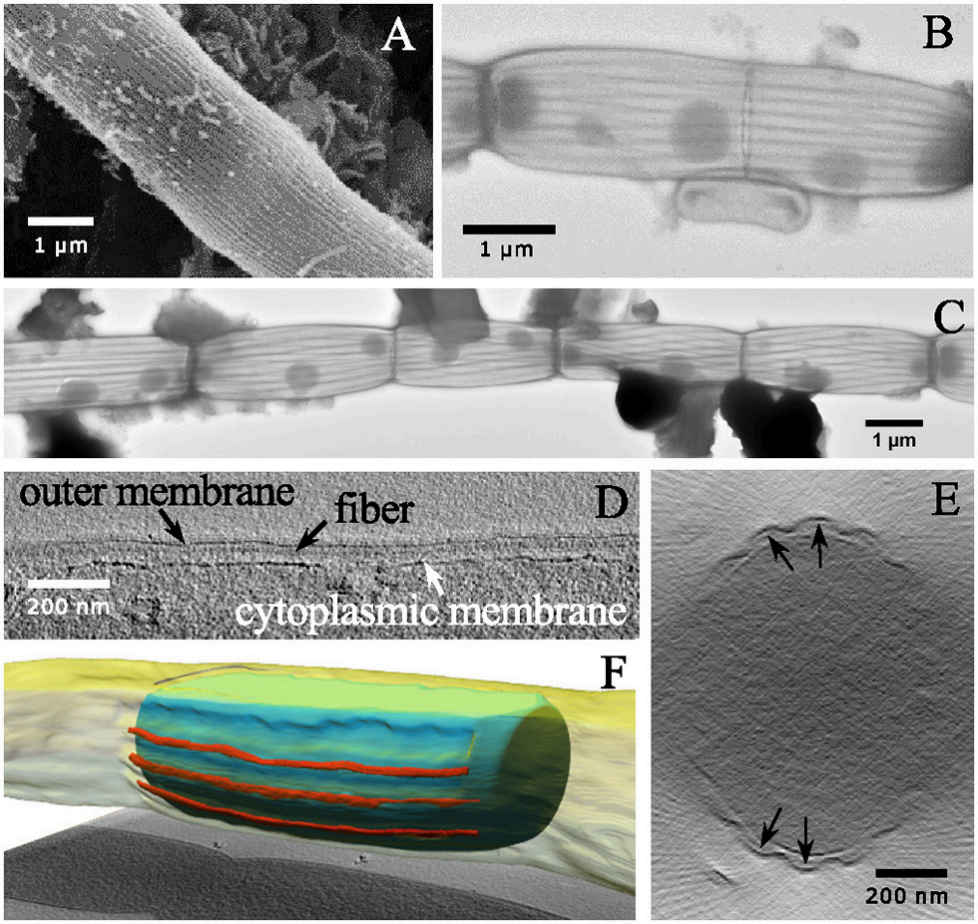
Electron micrographs of individual cable bacterium filaments. **(A)** Scanning electron microscopy image (20,000× 30 kV) of an individual cable bacterium filament after ethanol dehydration and critical-point drying. The cell surface shows the characteristic pattern of parallel ridges. **(B)** Transmission electron microscopy (TEM) image of a washed filament stained with uranyl acetate (11,000× 80 kV). The middle part shows the process of cell division. **(C)** Composite TEM image (11,000× 80 kV) of a string of uranyl acetate stained cells. The dark line pattern reveals how the cell envelope is segmented into parallel structural components. The dark circular inclusions in the cytoplasm are most likely intracellular polyphosphate accumulations. **(D)** A longitudinal tomographic slice through the cell envelope of a cable bacterium has been captured using cryo-electron tomography and shows fibers (black arrows) between the inner and outer membrane. **(E)** A transverse tomographic slice shows fibers positioned between the inner and the outer membrane (indicated by black arrows). **(F)** Reconstructed surface rendering of the periplasmic fibers (red), the inner membrane (blue) and outer membrane (yellow). Origin filaments: A, D-F Lake Grevelingen; B,C, Aarhus bay.

The SEM images confirmed that the surface of cable bacterium filaments has a conspicuous ultrastructure, as already previously observed (Pfeffer et al., 2012; Malkin et al., 2014). When cable bacterium filaments were dehydrated in an ethanol series and subsequently critically point dried with carbon dioxide, they retained a rounded cylindrical shape (Figure 1A). The cell surface showed a pattern of parallel ridges and grooves that runs along the entire longitudinal axis of every filaments. The ridges were continuous across cell junctions and no branching was observed. Across all geographical locations sampled and imaged, we observed that filaments can greatly vary in both diameter as well as in the numbers of parallel segments that were incorporated in the cell envelope. The diameter (*d*_*ESD*_) varied between 0.63 and 2.40 μm and the number of parallel ridge structures (*N*_*R*_*)* between 15 and 54. However, ridge dimensions remained similar across cable bacterium filaments providing a mean ridge width of δ_*R*_ = 144 ± 30 nm across all specimens investigated (*n* = 25). Given the large morphological heterogeneity of filaments investigated (great differences in both diameter and ridge number), and knowing that variation can be introduced during sample preparation, the observed variability in the ridge width is small.

When cable bacteria were exposed to consecutive washes in MilliQ water and subsequently air-dried, this generally led to “deflated filaments.” These are filaments with an apparent intact cell envelope, but which may have lost part of the cytoplasmic cell content. MilliQ is hypotonic in relation to the cytoplasm of the cell and results in an osmotic uptake of water by the cell, potentially leading to cell lysis. TEM images of MilliQ-washed and air-dried filaments (Figures 1B,C) generally showed a lack of electron adsorbing features inside the cell, suggesting a depleted cell content that allows the TEM electron beam to pass through. Instead, organic debris was often noted clinging to the cell surface, which may be expelled cytoplasmic material (Figure 1C). However, not all of the cell content was lost, as cells still showed a number of conspicuous, darkly stained globular inclusions. These globules are likely polyphosphate inclusions, which can accumulate intracellularly in the cytoplasm, and were previously identified by nanoscale secondary ion mass spectrometry analysis (Sulu-Gambari et al., 2016). Note that polyphosphate inclusions appear to be preferentially located near the cell junctions, although large variability exists with respect to their number and positioning inside the cells.

The MilliQ washing and air-drying procedure had little effect on the cell envelope, and so the deflation must happen in a somewhat gentle way, as we did not see many cells with clear damage from the lysis or a rupture of the cell envelope. In the majority of cases, the filamentous structure of the cable bacteria remained intact (Figure 1C). The observation that cable bacterium filaments retain their filament integrity after the MilliQ washing procedure, indicates that they possess a sturdy and resilient cell envelope structure, which can withstand sizeable osmotic forces. Instead of having a rounded, cylindrical shape, the MilliQ-washed and air-dried filaments were flatter, as the upper side of the cell envelope was collapsed onto the lower side. As a result, the filaments became wider in the middle of cells compared to the cell junctions (Figures 1B,C), which suggests that cell junctions are made of a stiff material.

After staining with uranyl acetate, the TEM images of MilliQ-washed and air-dried filaments revealed a set of dark lines running in parallel along the longitudinal axis of the filaments (Figures 1B,C). Cells caught in the process of cell division (Figure 1B) showed that the dark lines remained continuous across cell-to-cell junctions. The nature of these darkly stained lines was difficult to ascertain from the TEM images alone. It could either be uranyl acetate stain that is retained within the grooves of the ridge compartment (not washed away), or alternatively, or it could be some fiber structure that is stained within each ridge compartment. CryoET however confirmed the latter hypothesis, and demonstrated that a fiber network was embedded in the cell envelope (Figures 1D–F). Although the resolution was low due to the limited tilting range, fiber structures were clearly identifiable as a distinct structure located in the periplasm between the cytoplasmic and periplasmic membrane (Figures 1D,E). A reconstructed 3D image revealed how the fibers run in parallel in the periplasm (Figure 1F). Because the CryoET method is a stain-free method, and the periplasmic fibers showed a low inherent contrast, it was not possible to estimate the fiber thickness from the CryoET images.

**Figure 2:**
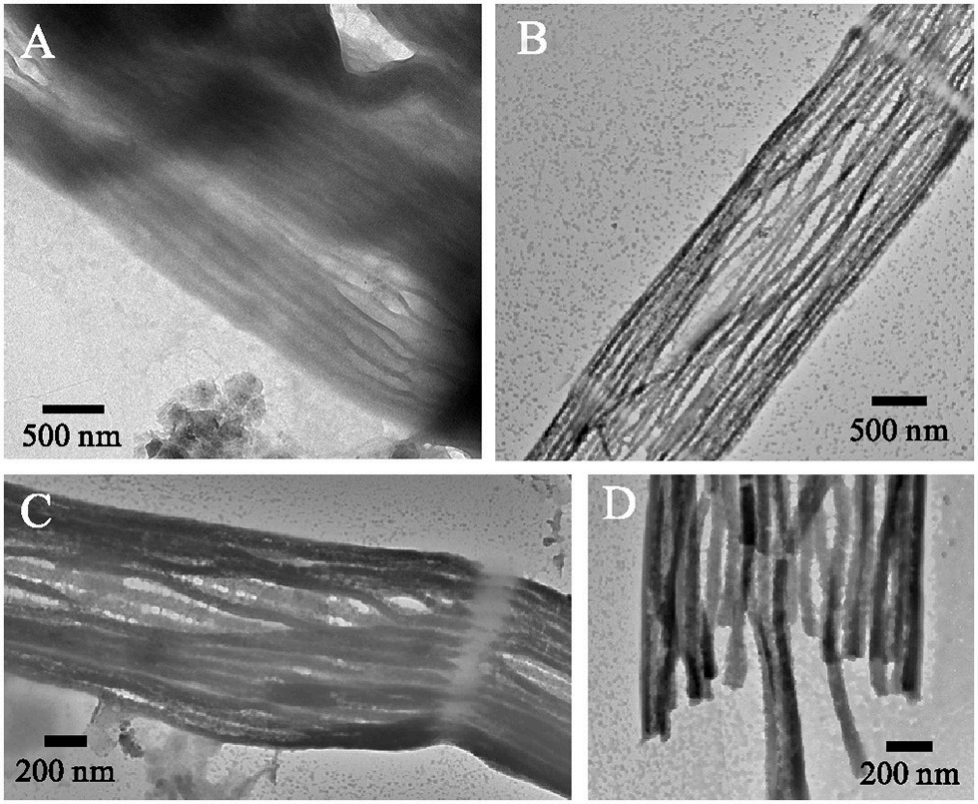
Fiber sheath structure. **(A)** Ruptured cell wall of a cable bacterium filament. Transmission electron microscopy (TEM) micrographs of unstained samples after air-drying. The cell envelope shows fibers that are visible as parallel cylinders. Their dark appearance suggests that the fibers consist of an electron dense material. **(B–D)** TEM images of the air dried remnants of a cable bacterium filament showing that a fiber cage structure is retained when cytoplasm and membranes have been removed. Origin filaments: A, Rattekaai Salt Marsh, B–D, Aarhus University lake.

Partially damaged filaments were occasionally encountered during SEM and TEM imaging. This enabled further insight into the structure of the cell envelope and the geometry of the periplasmic fiber network. TEM imaging of MilliQ-washed, air-dried, and unstained filaments with a partially ruptured cell envelope revealed clearly delineated fibers (Figure 2A). These fibers were cylindrical and had a dark appearance, which suggests that they were made of an electron-dense material that was distinct from the rest of the periplasm. Image analysis revealed a mean fiber diameter of 50 ± 7 nm (*n* = 20). In another instance, “ghost filaments” were extracted from the sediment enrichment, which had entirely lost all cell membrane and cytoplasm material (Figures 2B–D). The degradation process that gave rise to these ghost filaments was unclear, but a cage of parallel fibers remained, which were tightly connected by a ring structure at the cell junctions. Outside of the cell junctions, adjacent fibers appeared to be kept together with regularly spaced, small interconnections (Figure 2C). Image analysis on three separate ghost filaments provided a mean fiber diameter of 58 ± 12 (*n* = 8), 67 ± 7 (*n* = 15), and 46 ± 9 (*n* = 20) nm.

The above results are all based on SEM and TEM images of cable bacterium filaments that were collected—sometimes opportunistically—from different natural sediments at different instances. Although these filaments were all positively identified as cable bacteria, because they showed the characteristic ridge pattern on their outer surface, they could belong to different genotypes, or they could be in a different growth phase. To enable a more systematic analysis, we performed a detailed and quantitative investigation of filaments that were enriched in the same way from a single site (Rattekaai Salt Marsh, NL), thus reducing potential sources of morphological variation. Still, these sediment enrichments from a single site still showed filament diversity. Two different morphotypes were repeatedly retrieved from the same cores, indicating they were co-existing within the sediment enrichment: thin filaments with a diameter of ~0.7 μm and thick filaments with a diameter of ~3.5 μm. These two morphotypes were further investigated in detail with FIB-SEM and AFM to arrive at structures and dimensions of the cell envelope components.

### FIB-SEM Investigation of Intact, Thick Filaments

**Figure 3:**
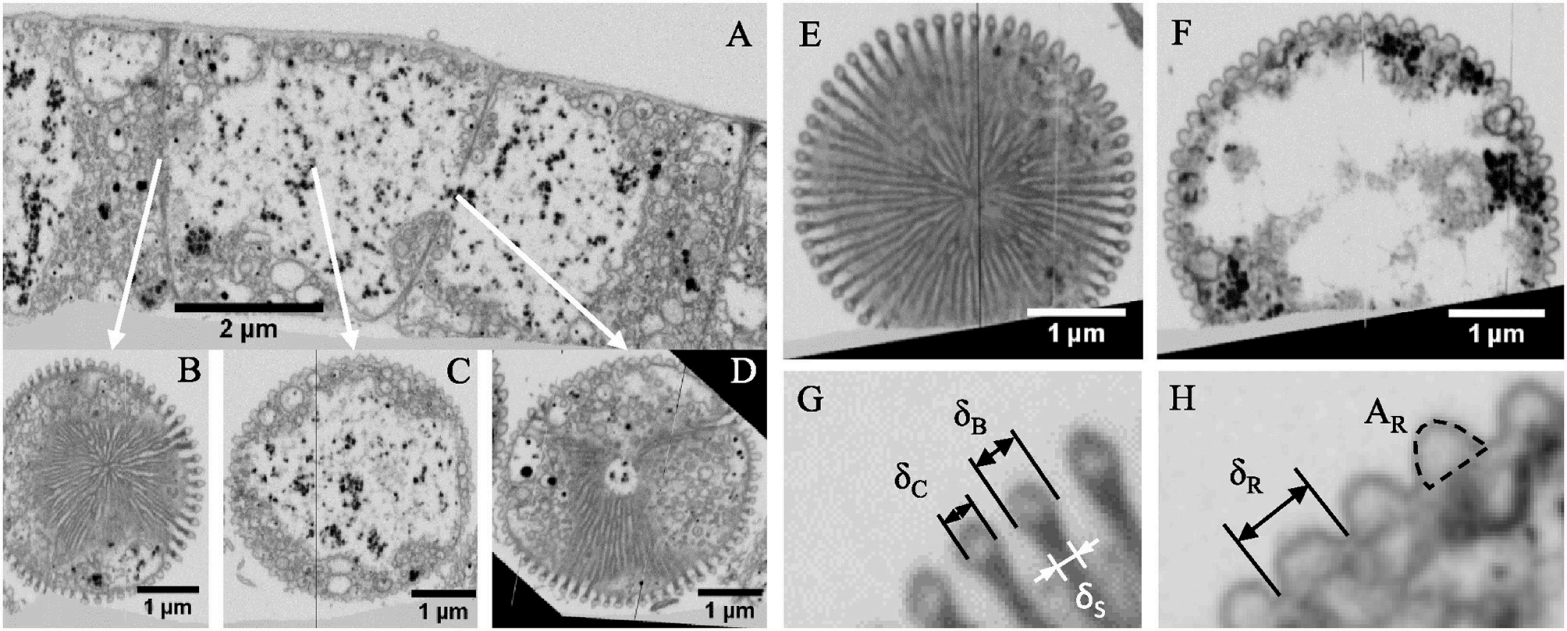
Internal structure of intact thick cable bacterium filaments. Specimens embedded in resin, stained with osmium tetroxide and uranyl acetate and imaged with focused ion beam—scanning electron microscopy. **(A)** A longitudinal cross-section with arrows indicating the cross sections. Dark granules range in diameter from 40 to 100 nm. Transverse cross-section **(B)** at a junction, exposing the cartwheel structure, **(C)** the middle of a cell, where the 61 ridge compartments continue from junction to junction along the cell envelope, and **(D)** a newly forming junction. A second filament showing: **(E)** a transverse cross-section exposing a full junction structure, and **(F)** a cross-section in the middle of a cell. **(G,H)** indicate how average dimensions of the bulb [bulb diameter (δ_B_), bulk core diameter (δ_C_), and stalk diameter (δ_S_)], and ridges [ridge width (δ_R_), and ridge compartment area (A_R_)], respectively, were measured. Origin filaments: Rattekaai Salt Marsh.

FIB-SEM images of MilliQ-washed, resin-embedded, thick filaments revealed additional details on the 3D structures of the ridge compartments and the cell-to-cell junctions (Figure 3). OsO_4_, which was used for fixation and optical contrast, stained the lipids of the inner and outer cell membranes, as well as vesicular structures within the cytoplasm. These vesicles were present in different sizes, and tended to be distributed near the periphery of the cell (Figures 3A,C). It is unclear whether the vesicle formation is an artifact of the sample preparation, or whether the vesicles truly serve a —presently unknown—function within the cells. The inner part of the cytoplasm showed large, unstained patches (white areas) that contained sparsely distributed thin, gray filaments (potentially DNA or parts of the cytoskeleton; Shih and Rothfield, 2006) and darkly stained granules (30–100 nm in size, resembling clusters of ribosomes). The unstained patches could be due to partial lysis during washing, problems with chemical fixation, or due to a reduced penetration of the OsO_4_ and uranyl acetate stains through the thick cell envelope.

In transverse cross-sections, the individual ridge compartments were clearly identifiable. Outside of the cell junctions, the ridge compartments had a half-circular shape, with a darkly stained outer lining, and a lighter, unstained core area (Figures 3C,F,H). FIB-SEM imaging revealed that the cell junctions have a conspicuous internal structure looking like a cartwheel (Figures 3B,D,E), where darkly stained “spokes” apparently radially from a central node (Figure 3E). While radiating outwards, the spokes bifurcate, thus augmenting the number of spokes. Eventually, each spoke terminates at a ridge compartment, which sits as a “bulb” upon a “stalk.” The spokes were stained in the same way as the lining of the ridge compartments, but the inner core of the terminal bulb was much less stained. A cell caught in the process of cell division (Figure 3D) suggests that the cartwheel structure is actually formed by an invagination and coalescence of the outer envelope during cell division. A step-by-step analysis of consecutive FIB-SEM images (Supplementary Video S1) confirmed that the fiber structures in the core of the ridge compartment remain continuous across the cell junctions.

Detailed image analysis of the FIB-SEM data was done on two separate filaments. The first filament (BF1; Figures 3A–D) had 61 ridges (*N*_*R*_) and a perimeter of 12.5 ± 0.2 μm, and so we obtained a diameter of 4.0 ± 0.1 μm and a mean ridge width of 205 ± 3 nm. The second filament (BF2; Figures 3E–H) had fewer ridges (*N*_*R*_ = 58), but also a slightly smaller cell diameter (3.9 ± 0.1 μm), thus providing a similar mean ridge width of 213 ± 5 nm. The ridge compartments adopted a half-circular shape in the middle of the cells. The cross-sectional area of a ridge compartment (which includes the dark linings) was comparable for both filaments (11 ± 3 × 10^3^ nm2 for BF1 and 16 ± 2 × 10^3^ nm2 for BF2; Table 1). The geometry of the “stalked bulb” structures at the cell junctions was highly similar in both filaments. The diameter of the terminal bulb was 124 ± 13 nm in BF1 and 117 ± 10 nm in BF2 (Table 1). The diameter of the faintly stained inner core of the terminal bulb (57 ± 8 nm and 46 ± 6 nm in BF1 and BF2, respectively) corresponded very well with the fiber diameter previously documented from the TEM images of the ghost filaments (Figure 2), suggesting that both could be the same structure. The circular terminal bulb had a cross-sectional area of 12 ± 3 × 10^3^ nm2 in BF1 and 11 ± 2 × 10^3^ nm2 in BF2, which coincided with the areas of half-circular ridges in the middle of the cells. This suggests that the ridge compartments changed shape at the cell junctions (from half-circular to circular), but contained about the same amount of periplasmic material. The width of the stalks in the cartwheel structure was 82 ± 14 nm in BF1 and 59 ± 9 nm in BF2, which is too broad to only represent two stacked membranes, indicating that the stalks must include other material in addition to membrane lipids.

**Table 1:** Geometrical parameters for cable bacterium filaments obtained from detailed image analysis of FIB-SEM data.

**Parameter**	**Symbol**	**Units**	**BF1**	**BF2**	**SF1**	**SF2**	**EF1**	**EF2**
Perimeter	*P*	μm	12.5 ± 0.2	12.3 ± 0.3	3.5 ± 0.3	2.0 ± 0.1	7.4 ± 0.6	7.3 ± 0.6
Diameter^*^	*d_*ESD*_*	μm	4.0 ± 0.1	3.9 ± 0.1	1.1 ± 0.1	0.6 ± 0.1	2.4 ± 0.2	2.3 ± 0.2
Ridge number	*N_*R*_*	-	61	58	15	16	62	52
Ridge width^*^	*δ_*R*_*	nm	205 ± 3	213 ± 5	231 ± 21	126 ± 6	120 ± 10	140 ± 12
Ridge compartment area	*A_*R*_*	10^3^ nm^2^	11 ± 3	16 ± 2	9 ± 1	5 ± 1	NA	NA
Bulb diameter	*δ_*B*_*	nm	124 ± 13	117 ± 10	103 ± 13	111 ± 22	86 ± 13	92 ± 12
Bulb area^*^	*A_*B*_*	10^3^ nm^2^	12 ± 3	11 ± 2	8 ± 4	10 ± 8	6 ± 2	7 ± 2
Bulb core diameter	*δ_*C*_*	nm	57 ± 8	46 ± 6	NA	NA	24 ± 8	NA
Stalk width	*δ_*S*_*	nm	82 ± 14	59 ± 9	68 ± 7	49 ± 5	63 ± 11	48 ± 8

Codes: BF, intact, thick filament; SF = intact, thin filament; EF = extracted, thick filament. Parameters marked with

* *are calculated from other parameters*.

### AFM Investigation of Intact, Thick Filaments

**Figure 4:**
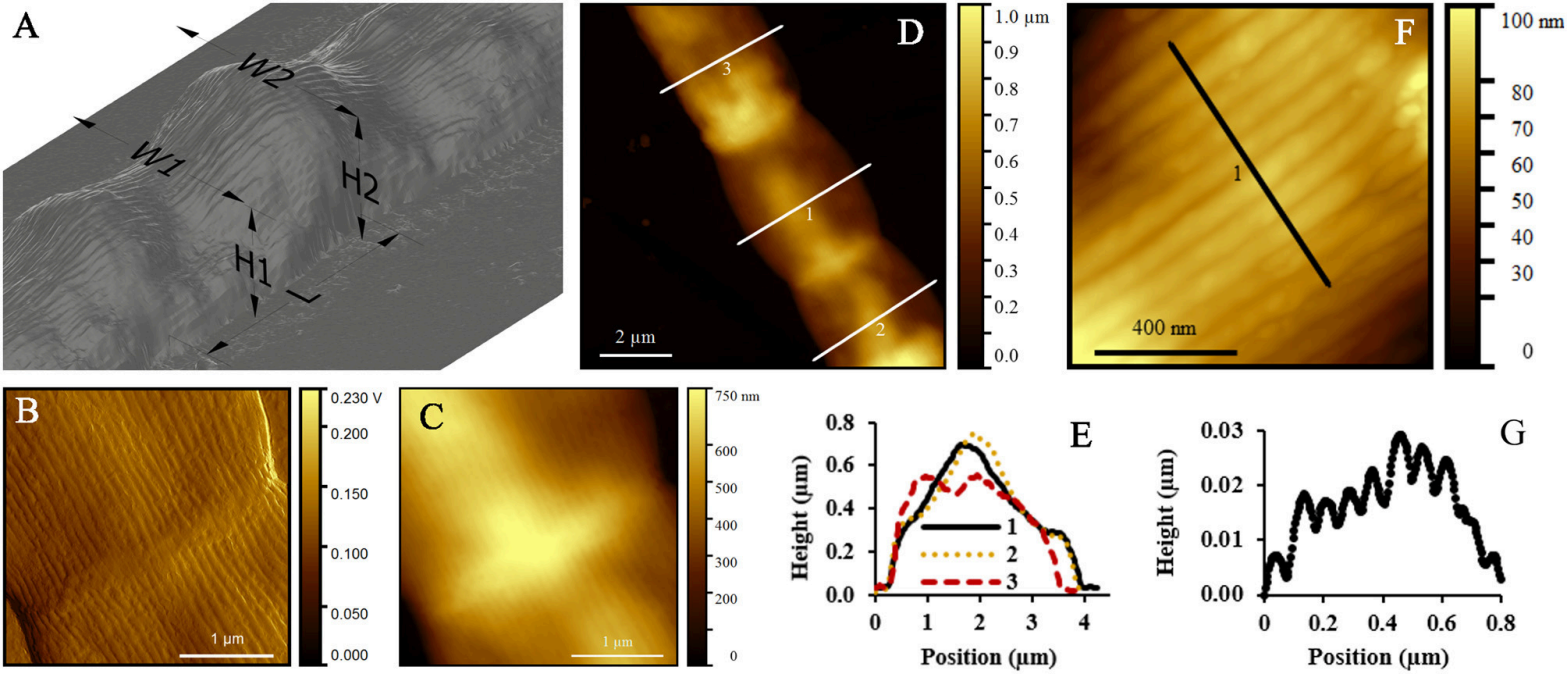
Atomic force microscopy (AFM) study of intact cable bacterium filaments. **(A)** 3D representation of a typical intact filament, deposited in MilliQ and left to dry in air, obtained from AFM. **(B)** Peak force error and **(C)** topography images with ridges running over the longitudinal axis of the filament. **(D)** AFM topography image of three cells of one filament, showing a variation in height profiles given in **(E)**. **(F)** AFM topography image of a detail of one cell, where the separate ridges are clearly visible. **(G)** Height profile as indicated in **(F)**, showing the height (in μm) of multiple ridges. Origin filaments: Rattekaai Salt Marsh.

The geometry of thick filaments was additionally characterized by AFM topography imaging after MilliQ-washed filaments were air dried on mica substrates (Figure 4A). Individual cells of these intact, thick filaments had an average length of 4.93 ± 1.28 μm. Peak force error measurements are optimal for detection of sudden changes in the height, stiffness, adhesion or other material properties. As a result, they provided an improved contrast image of the detailed ridge-structure of cable bacteria (Figure 4B) compared to standard topography images (Figure 4C).

When air-dried in preparation for AFM, the filaments substantially deflated due to the lack of chemical or physical fixation (as previously seen in the TEM images). Height profiles taken across the middle of the cells revealed substantial variability in height profiles (Figures 4D,E). Overall we found that the measured width of an intact filament (3.71 ± 0.61 μm, Table 2) was more than four times the measured height (0.87 ± 0.14 μm) at the middle of cells. In contrast, at the junctions, the filaments were 11% less wide (3.38 ± 0.44 μm), but 30% higher (1.13 ± 0.18 μm), thus revealing that the junctions had a more rigid structure that was more resistant to deflation. The ridge compartments were detectable as protrusions in the AFM topography images (Figures 4F,G). The ridge compartment width was on average 0.10 ± 0.02 μm (Figure 4G). The diameter of the thick intact filaments as determined from AFM data (2.50 ± 0.51 μm) is 37% smaller compared to the diameter obtained from FIB-SEM data (3.9–4.0 μm).

**Table 2:** Geometrical parameters for cable bacterium filaments as obtained from detailed analysis of AFM data.

**Parameter**	**Symbol (μm)**	**BF**		**SF**		**EF**
Cell length	*L*	4.93 ± 1.28	*N* = 13	2.22 ± 0.63	*N* = 14	3.85 ± 0.90	*N* = 62
Width middle cell	*W1*	3.71 ± 0.61	*N* = 67	0.85 ± 0.13	*N* = 18	3.78 ± 0.44	*N* = 53
Width junction	*W2*	3.38 ± 0.44	*N* = 63	0.66 ± 0.06	*N* = 17	3.05 ± 0.41	*N* = 61
Height middle cell	*H1*	0.87 ± 0.14	*N* = 67	0.19 ± 0.04	*N* = 18	0.12 ± 0.05	*N* = 53
Height junction	*H2*	1.13 ± 0.18	*N* = 63	0.24 ± 0.02	*N* = 17	0.32 ± 0.11	*N* = 61
Ridge width	*δ_*R*_*	0.10 ± 0.02	*N* = 13	0.10 ± 0.01	*N* = 4	0.14 ± 0.03	*N* = 19
Perimeter^*^	*P*	7.85 ± 1.59	*N* = 67	1.56 ± 0.20	*N* = 18	6.27 ± 1.49	*N* = 53
Diameter^*^	*d_*ESD*_*	2.50 ± 0.51	*N* = 67	0.50 ± 0.06	*N* = 18	1.99 ± 0.47	*N* = 53

The dimensions (W, H, and L) are illustrated in Figures 4, 7. Codes: BF = intact, thick filaments; SF = intact, thin filaments; EF = extracted, thick filaments. Parameters marked with

* *are calculated from other parameters. N denotes the number of filaments measured*.

### FIB-SEM and AFM Investigation of Thin Filaments

**Figure 5:**
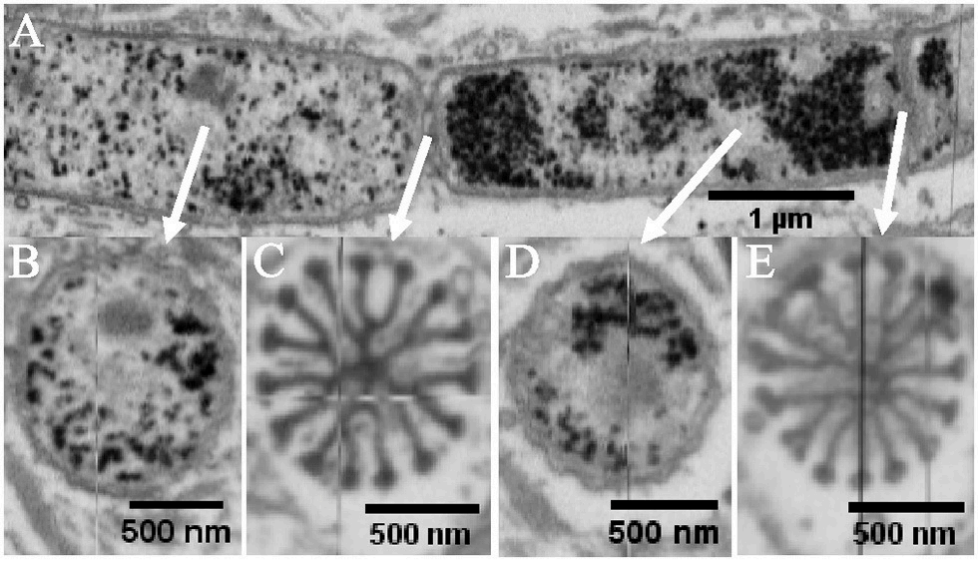
Internal structure of intact thin cable bacterium filaments. Specimens embedded in resin, stained with osmium tetroxide and uranyl acetate and imaged with focused ion beam—scanning electron microscopy. **(A)** A longitudinal cross-section of a filament with a diameter of 1.2 μm is shown, with arrows indicating the location of transverse cross-sections. Dark granules range in diameter from 40 to 60 nm. Transverse cross-sections are shown for **(B,D)** at the middle of a cell, and **(C,E)** at a junction, exposing the cartwheel structure. In **(B**–**E)**, 15 ridge compartments continue from one cell to the next while forming the typical bulbs at the cell junctions. Origin filaments: Rattekaai Salt Marsh.

To enable a comparison of differently sized cable bacterium filaments, we also investigated thin filaments, which were found in the same enrichments together with the thick filaments. FIB-SEM data revealed that also the thinner filaments incorporated darkly stained granules in the cytoplasm. They also displayed the same cartwheel structure at the cell junctions, as observed in the thicker filaments. Detailed image analysis was done on two separate thin filaments (Figure 5 and Table 1). One filament (SF1, Figures 5A–E) had 15 ridges and a cell diameter of 1.1 ± 0.1 μm. Despite its smaller size, the resulting ridge width (231 ± 21 nm) was similar to the thick filaments, while the ridge compartment area was slightly smaller [(9 ± 1) × 10^3^ nm2]. At the cell junctions, the geometry of the stalked bulb was highly similar to that of the thick filaments (δ_B_ = 103 ± 13 nm, A_B_ = 8 ± 4 × 10^3^ nm2 and δ_S_ = 68 ± 7 nm). However, this time, the inner core of the terminal bulb did not stain differently from the surrounding material (Figures 5C,E). The second thin filament (SF2; image not shown) had 16 ridges, but a smaller cell diameter of 0.6 ± 0.1 μm, leading to a smaller ridge width of 126 ± 6 nm, and a smaller ridge area of (5 ± 1) × 10^3^ nm2. Still, the geometry of the terminal bulbs of the cartwheel structure at the cell junctions remained similar to that of the other filaments investigated (δ_B_ = 111 ± 22 nm, A_B_ = (10 ± 8) × 10^3^ nm2, and δ_S_ = 49 ± 5 nm).

AFM imaging of MilliQ-washed, air-dried, thin filaments revealed an average cell length of 2.22 ± 0.63 μm. Similar to the thick filaments, the middle of the cell deflated more compared to the cell junctions (Table 2). Moreover, the ESD as measured around the middle of the cells was 0.50 ± 0.06 μm, which was again lower than obtained for resin-embedded cells, thus confirming that cells were shrinking during the process of air-drying. Despite the great difference in morphology between thick and thin filaments, the ridge width at the junctions based on the AFM topography imaging was highly similar (δ_R_ = 0.10 ± 0.01 μm; Table 2).

### Investigation of Extracted Filaments

**Figure 6:**
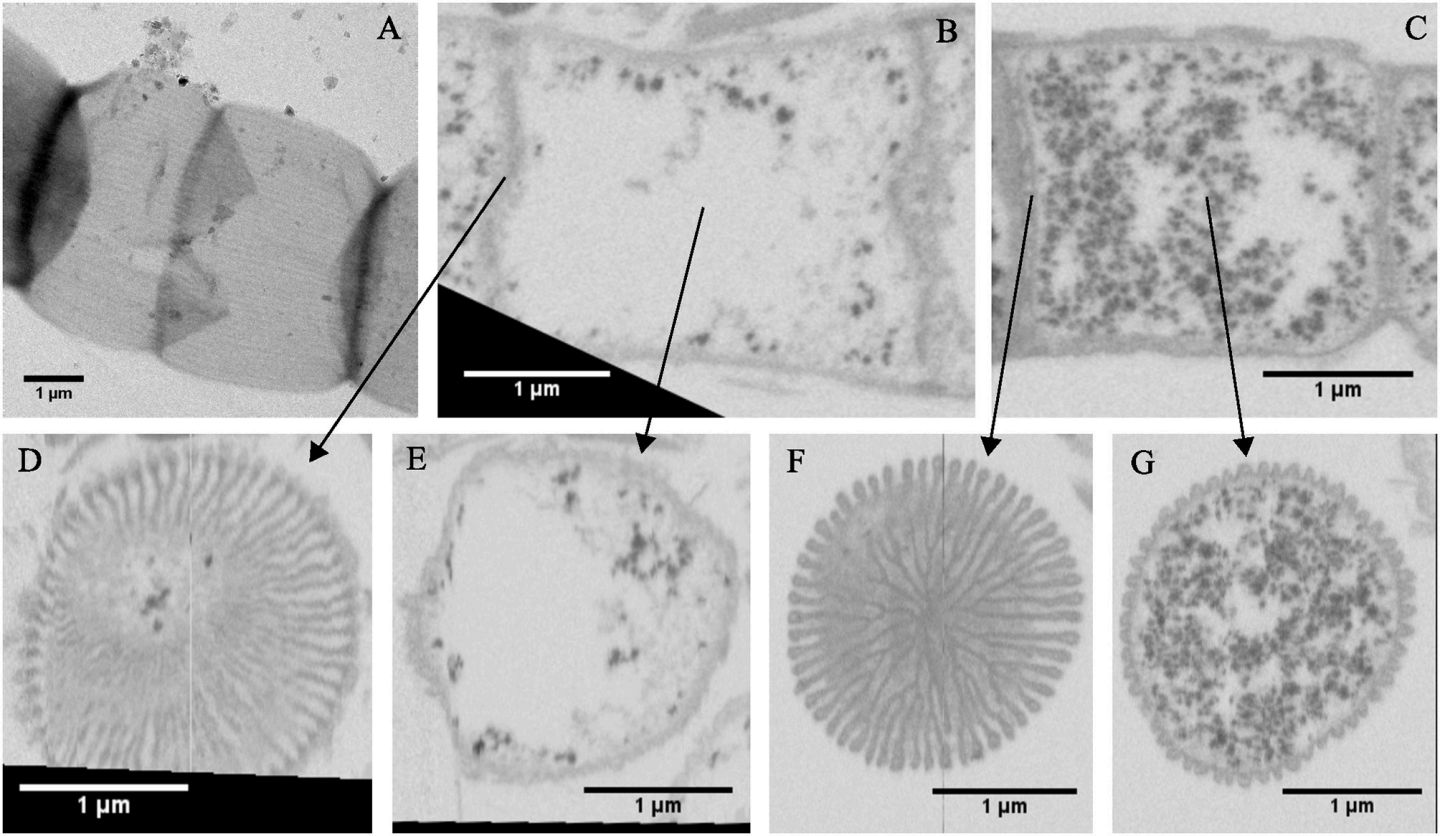
Structure of thick filaments after fiber sheath extraction (SDS+EDTA). Filaments treated with an SDS+EDTA protocol and imaged with transmission electron microscopy (TEM) and focused ion beam—scanning electron microscopy (FIB-SEM). **(A)** Unstained TEM image (120 kV) showing the fibers connected at the junctions. **(B)** FIB-SEM longitudinal cross-section of an extracted filament with arrows indicating the location of the transverse cross-sections shown in **(D,E)**. Only a few granules remain, their diameter ranges from 40 to 90 nm. The middle of a cell **(E)** contains much less material after extraction, but the cartwheel structure **(D)** remains intact at the junctions. A second extracted filament **(C)** retains more material in the cytoplasm, but also here the cartwheel structure remains intact as can be seen in the transverse cross-sections shown in **(F,G)**. Origin filaments: Rattekaai Salt Marsh.

An SDS+EDTA extraction of cable bacterium filaments was developed with the objective of removing the membranes and cytoplasm and retaining the periplasmic fiber network structure. TEM imaging revealed that cable bacteria retain their long filamentous form after extraction. A cylindrical sheath structure remained, where sufficient material had been removed for the electron beam of the TEM to pass through (Figure 6A). Even without staining, the sheath structure showed a light-dark striped pattern (Figure 6A), where the darker electron-dense lines likely corresponded to the periplasmic fibers (see also Figure 2A). The ring-shaped cell junctions remained much darker (Figure 6A), and they were also severely constricted compared to the middle part of the cells, suggesting that the internal “cartwheel ”structure largely remains present after extraction and restricts the junctions from flattening. FIB-SEM imaging provided additional details on the periplasmic fiber sheath structure (Figures 6B–G). After extraction, the cytoplasm lost nearly all of its internal structures, apart from some darkly stained small granules that remained dispersed in the cell space (Figures 6B,E). As also observed in the TEM image (Figure 6A), the ridge pattern remained intact, but the dark lining of the ridge compartments was gone, suggesting that the outer and cell lipid membrane were removed. Remarkably, the cartwheel structure at the cell junctions remained intact after extraction (Figures 6D,F), indicating that it is highly chemically resistant. As SDS removes lipid membranes, the spokes of the cartwheel cannot solely consist of double-folded lipid membranes, but must contain additional material. As seen for the intact filaments, the stalked bulbs contained a core that is more lightly stained (Figure 6F).

Detailed analysis of the FIB-SEM images was carried out for two extracted filaments. One filament (EF1, Figures 6B,D,E) had 62 ridges and diameter of 2.4 ± 0.2 μm, providing a ridge width of 120 ± 10 nm. The other filament (EF2, Figures 6C,F,G) had 52 ridges and a cell diameter of 2.3 ± 0.2 μm, providing a ridge width of 140 ± 12 nm. The average ridge area could not be determined for EF1 or EF2, as the individual ridge compartments could not be clearly distinguished in the middle of the cells. The geometry of the bulb and stalks was highly comparable to the intact filaments suggesting that little material had been removed (Table 1).

**Figure 7:**
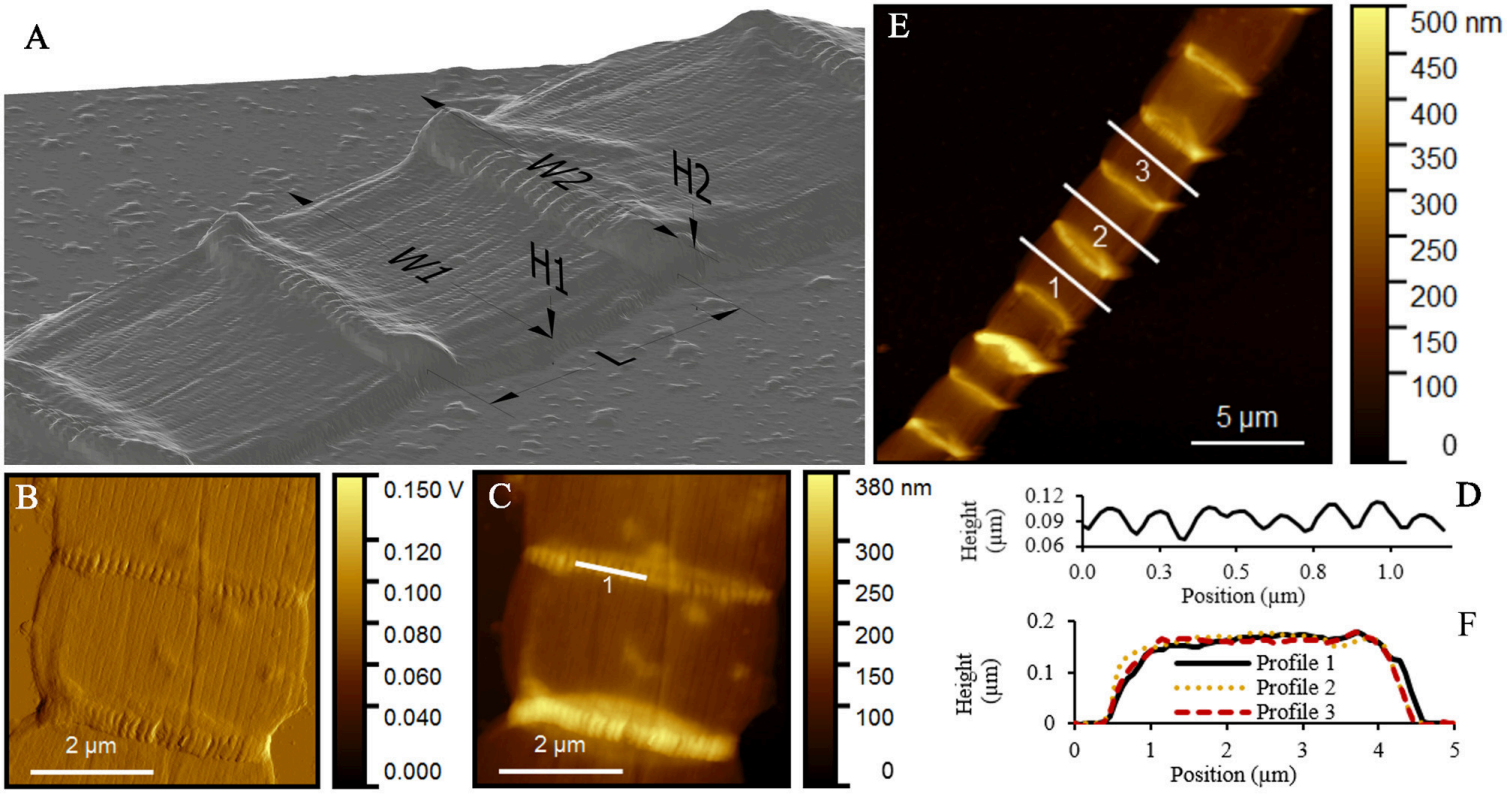
Atomic force microscopy (AFM) study of extracted cable bacterium filaments. **(A)** 3D-representation of a typical extracted filament, which was deposited in MilliQ after extraction and left to dry in air for AFM. **(B)** Peak force error and **(C)** topography images with ridges running over the longitudinal axis of the cell. **(D)** Height profile across a junction as indicated in **(C)** showing the individual ridges at a junction. **(E)** AFM topography image of nine cells of one filament, with typical height profiles shown in **(F)**. Origin filaments: Rattekaai Salt Marsh.

In the AFM images, the extracted filaments had a similar width as the intact filaments, but the overall height of the extracted filament was substantially lower (Table 2). The cells had a generally flat appearance, and the junctions clearly stood out and attained a height that was nearly three times higher (320 ± 110 nm) than in the middle of the cells (120 ± 50 nm) (Figures 7A,E,F). The extracted filaments represented the two cell envelope sheaths that collapsed on top of each other in the middle of the cell, implying that the height of one such sheath was ~60 nm, which is only slightly higher than the diameter of a periplasmic fiber (~50 nm; as determined from Figure 2). This demonstrated that substantial material had been removed in the extraction process. Still, the parallel ridge compartments were identifiable from each other (Figures 7B,C), and were separated from each other by a valley of ~30 nm depth (Figure 7D). The ridge compartments in the extracted filaments (140 ± 30 nm) were wider compared to the intact filaments.

## Discussion

The parallel ridge pattern endows the cell envelope of cable bacteria with a distinct structure, not previously observed in filamentous microorganisms (Figures 1, 4, 7). Our data revealed that individual ridge compartments have similar dimensions in cable bacterium filaments that greatly differ in diameter. Cable bacteria hence appear to build their cell envelope by the parallel concatenation of a standard type of ridge compartment. Our FIB-SEM data confirmed that the width of a ridge compartment was similar (205–231 nm) between thick and thin filaments (Table 1). In all FIB-SEM data, there was one exception of a small filament, where the ridge compartment was smaller (126 nm). Yet, our AFM data additionally confirmed that the ridge width is highly uniform in thick and thin filaments (Table 2). Larger diameter filaments hence simply incorporate more parallel ridge compartments.

**Figure 8:**
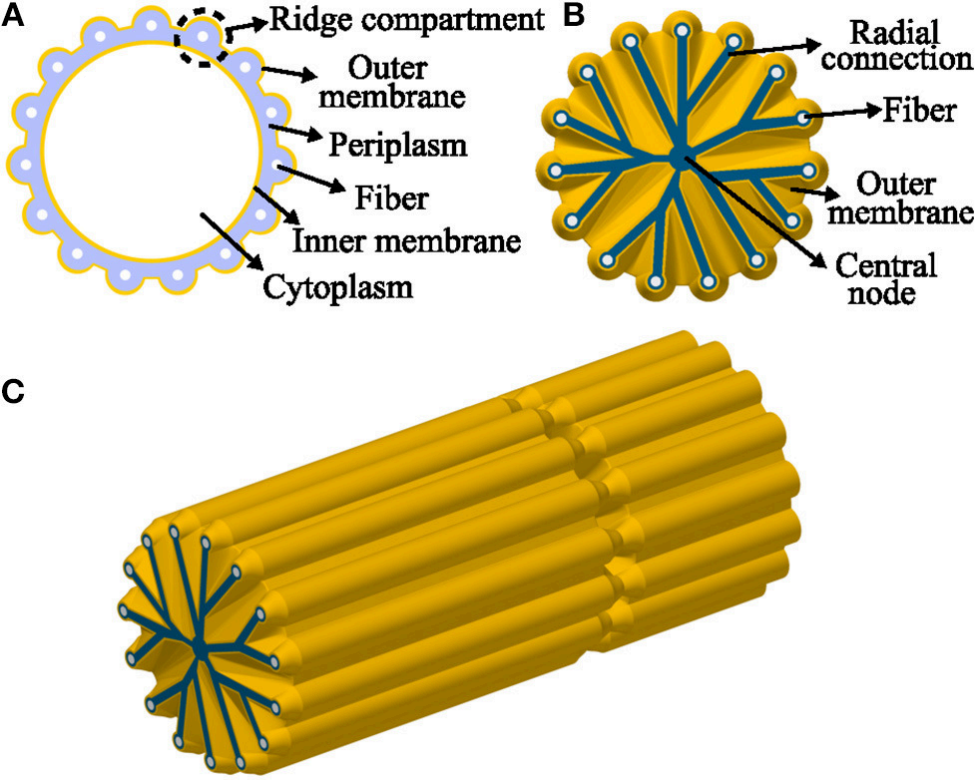
Model representation of a cable bacteria cell. **(A)** Model transverse cross-section of a cable bacterium cell, showing the outer membrane (ridges) and the fibers in the periplasm. **(B)** Model transverse cross-section at a junction, the outer membrane folds in toward the central node, surrounding unknown radial connections. **(C)** 3D-view of one and a half cell. Starting at a junction and ending at the middle of a cell, the fibers continue along the length of the filament.

The ridge width in the AFM data (~100 nm) was significantly smaller (*P* < 0.05, *t*-test) than in the FIB-SEM data (~210 nm) most likely due to shrinkage of the filaments resulting from air-drying prior to AFM imaging. Overall, the comparison of corresponding dimensions between AFM (non-fixed, air-dried filaments; Table 2) and FIB-SEM (chemically fixed and resin-embedded filaments; Table 1) revealed that sample preparation can have a large effect on the geometry of the filaments. The dimensions derived from the FIB-SEM data were used for construction of a 3D model of a cable bacterium filament (Figure 8), based on the dimensions of filament SF1.

A surprising observation was the high variability in the number of ridges that were incorporated by cable bacterium filaments retrieved from the same sediment enrichment. The six filaments that were examined with FIB-SEM turned out to have all a different number of ridges (thick filaments: 52, 58, 61, 62 and thin filaments: 15, 16). All these filaments were retrieved from the same small parcel of sediment in the same sediment enrichment core. Accordingly they were co-existing with each other at the same time and place. This begs an intriguing question about the relationship between genotype and phenotype. Are the different ridge numbers an exponent of genotypic variation, or do cable bacteria species simply show a large amount of phenotypic variation? At present, the genetic variation in cable bacteria has only been assessed in one study (Trojan et al., 2016), which revealed six different species distributed over two separate genera (*Ca*. Electrothrix and *Ca*. Electronema). Database searches furthermore revealed 185 16S rRNA gene sequences that affiliated with cable bacteria, of which 120 sequences could be assigned to one of the six candidate species, while the remaining 65 sequences indicated the existence of up to five additional species (Trojan et al., 2016). Although the genetic diversity of cable bacteria is at present likely underestimated, it is unlikely that six individual filaments picked from a sediment would belong to six different cable bacterium species. Therefore, our data seem to suggest that a large amount of intra-species phenotypic variation exists. One explanation could be that the cell diameter increases during the growth process and hence more ridges would be progressively included. Clearly, follow-up studies are needed to properly resolve the link between growth, morphology and phylogeny of cable bacteria.

FIB-SEM imaging revealed that the cell junctions of thick and thin filaments have a conspicuous “cartwheel” structure, where dark stained “spokes” converge to a central node (Figures 3, 5). Starting from a ridge compartment, the spokes merge while radiating inwards, thereby reducing the number of spokes until they terminate at the center of the cell junction. A cell caught in the process of division (Figure 3D) shed light on how this cartwheel structure is formed. In this process, the outer membrane first engulfs the fiber structures and then descends toward the central node. However, the width of the spokes of the cartwheel structure (60–80 nm) indicate that this structure is too wide to only represent two stacked membranes, and so the observed “stalks” must include other material in addition to membrane lipids.

When we combine the cryoEM (Figures 1D–F), TEM (Figure 2), and FIB-SEM (Figures 3E,G) data, we conclude that a fiber structure is located within the periplasmic space of each ridge compartment (Figure 2). These fiber structures were clearly identifiable as electron-dense, dark cylinders in TEM images from unstained samples (Figure 2A) and as fiber cages in ghost filaments (Figures 2B–D). An alternative hypothesis is that fiber structures are associated with cytoskeleton, which would imply that the fibers are not located in the periplasm, but lie underneath the cytoplasmic membrane. This idea is however not supported by our data, as our CryoEM images (Figures 1D–F) show an electron dense structure in the periplasm, and our FIB-SEM images (Figure 3) do not show any fiber structures underneath the cytoplasmic membrane. Instead, the FIB-SEM images reveal a fiber structure as a light patch within the center of the bulbs of the cell junctions (after specimens were stained with OsO_4_ and uranyl acetate). If we track this fiber structure through consecutive FIB-SEM images (Supplementary Video S1), then the fiber continues its way into the periplasm of the ridge compartments (Figure 3). Moreover, when membranes and cytoplasm are removed by a suitable extraction procedure, we only retain a periplasmic fiber sheath (Figures 6B–G), which still shows the characteristic pattern of electron-dense fibers (Figure 6A). Conjointly, the imagery data derived from cryoEM (Figures 1D–F), TEM (Figures 2, 6A) and FIB-SEM (Figures 3, 5, 6) are only consistent with a location of the fibers inside the periplasm.

The light appearance of the fiber structure in the FIB-SEM images suggests that the inner core area either forms a structure that is too dense for the stains to penetrate or that it consists of compounds with a low affinity for OsO_4_. The diameter of the fiber (~50 nm) was highly similar in TEM (Figure 2) and FIB-SEM images (Figure 3). In the CryoEM images (Figures 1D–F), the fibers appeared thinner, but here the fiber structure was poorly delineated, as the CryoEM images suffered from a low resolution and limited contrast, due to the limited tilting range. In cross-sections taken in the middle of the cell, the ridge compartments took a half-circular shape (Figure 3H), and the FIB-SEM data showed no distinct coloration of the fibers within the periplasmic space. However, 3D reconstruction of FIB-SEM images demonstrated that fibers are continuously running through the ridge compartments and are uninterrupted from cell to cell (Figures 3, 5, 6). Integrating all the available imaging data, we conclude that each ridge compartment does contain a fiber structure, but that additional work is needed to better understand the staining response of the fibers and surrounding periplasmic material in connection to the composition of these structures.

This raises the question about the functional role of the fiber network. One option is that the fibers provide rigidity and structural support to the long filaments that need to bridge centimeter distances in a dense sediment matrix characterized by substantial contact forces. Our microscopic observations reveal that when filaments are pulled out from the sediment, they can withstand a substantial stress before breaking, and thus cable bacterium filaments must possess a substantial tensile strength. A second proposition is that the fiber structures act as a conductive “wire network” enabling intercellular electron transport along the longitudinal axis of the bacterial filaments. Electrostatic force microscopy has previously revealed a distinct elevation of electrostatic force on the ridges as compared to the intermittent areas, indicating that the underlying fiber structures possess significant polarizability or charge storage capacity (Pfeffer et al., 2012). Overall, our data provide a first, quantitative structural model of the fiber network in the cell envelope of cable bacteria, but further studies are needed to determine the composition and electrical properties of the periplasmic fibers in order to resolve the mechanism of long-distance electron transfer in cable bacteria.

## Data Availability Statement

Partial datasets analyzed for this study are included in the manuscript and the supplementary files. The raw imaging data will be made available by the authors, without undue reservation, to any qualified researcher.

## Author Contributions

E-MZ, SH-M, JG, HB, and FM designed the filament extraction procedure. SH-M performed the filament extractions. E-MZ collected SEM images. AB, LD, TB, and JDH performed TEM imaging. FM did image analysis of SEM and TEM images. RK performed cryoEM imaging and 3D reconstruction. AK performed FIB-SEM imaging and RC performed the image analysis of FIB-SEM images. RC, JD, and RTE collected AFM imaging. RB and RC performed image analysis of AFM images assisted by RV and JM. RC and FM wrote the manuscript with input from all authors.

### Conflict of Interest Statement

The authors declare that the research was conducted in the absence of any commercial or financial relationships that could be construed as a potential conflict of interest.

## Supplementary Material

Supplementary Video S1 FIB-SEM data of filament BF2.
